# Determination of HPLC-UV Fingerprints of Spanish Paprika (*Capsicum annuum* L.) for Its Classification by Linear Discriminant Analysis

**DOI:** 10.3390/s18124479

**Published:** 2018-12-18

**Authors:** Xavier Cetó, Núria Serrano, Miriam Aragó, Alejandro Gámez, Miquel Esteban, José Manuel Díaz-Cruz, Oscar Núñez

**Affiliations:** 1 Department of Chemical Engineering and Analytical Chemistry, University of Barcelona, Martí i Franquès 1-11, E08028 Barcelona, Spain; xavier.ceto@ub.edu (X.C.); miriara2@hotmail.com (M.A.); alejandrogamezcr@gmail.com (A.G.); miquelestebanc@ub.edu (M.E.); josemanuel.diaz@ub.edu (J.M.D.-C.); oscar.nunez@ub.edu (O.N.); 2 Research Institute in Food Nutrition and Food Safety, University of Barcelona, Av. Prat de la Riba 171, Edifici Recerca (Gaudí), E-08901 Santa Coloma de Gramanet, Barcelona, Spain; 3 Serra Hunter Fellow, Generalitat de Catalunya, Spain

**Keywords:** HPLC-UV, Spanish paprika, polyphenols, Protected designation of origin, linear discriminant analysis, food authentication

## Abstract

The development of a simple HPLC-UV method towards the evaluation of Spanish paprika’s phenolic profile and their discrimination based on the former is reported herein. The approach is based on C_18_ reversed-phase chromatography to generate characteristic fingerprints, in combination with linear discriminant analysis (LDA) to achieve their classification. To this aim, chromatographic conditions were optimized so as to achieve the separation of major phenolic compounds already identified in paprika. Paprika samples were subjected to a sample extraction stage by sonication and centrifugation; extracting procedure and conditions were optimized to maximize the generation of enough discriminant fingerprints. Finally, chromatograms were baseline corrected, compressed employing fast Fourier transform (FFT), and then analyzed by means of principal component analysis (PCA) and LDA to carry out the classification of paprika samples. Under the developed procedure, a total of 96 paprika samples were analyzed, achieving a classification rate of 100% for the test subset (n = 25).

## 1. Introduction

The quality of natural food products is an issue of great interest in our society, especially when dealing with products with protected designation of origin (PDO). Several health-promoting properties of those have been partly attributed to the presence of bioactive compounds, which also play an important role in food sensorial and functional properties. Paprika, or chili pepper, which is a red seasoning powder with a characteristic flavor obtained from the drying and grinding of certain varieties of red peppers (*Capsicum annuum* L.), is one of the most widely used food colorants in both culinary and industrial applications. The intense and characteristic red color of paprika is due to its high carotenoid pigment content produced during fruit ripening, that apart from conferring color to food provides important health benefits [1,2,3,4]. Along with carotenoids, chlorophylls, tocophenols, capsaicinoids, and phenolic compounds are also important bioactive compounds present in paprika. The latter act as antioxidants, and are important in plant defense responses, having an impact in the resulting fruit quality [5].

Spain is one of the main paprika producing countries in Europe, where it is mainly cultivated in two regions: Murcia, with 21% of the country’s production in 2015 (1010 tones distributed over 381 ha) and Extremadura (district of La Vera, Cáceres) with 71% of total production (3362 tones distributed over 1241 ha) [6]. Both regions have been awarded a designation of origin: “Pimentón de La Vera” and “Pimentón de Murcia”, respectively. Murcia paprika is mainly produced from red peppers of the *Bola* variety, whereas La Vera paprika is produced from *Jaranda*, *Jariza*, *Jeromín,* and *Bola* red pepper varieties. Depending on the red pepper variety employed, three different paprika types are commercialized: sweet (*Bola* and *Jaranda* varieties), bittersweet (*Jariza* and *Jaranda* varieties) and spicy (*Jeromín*, *Jaranda,* and *Jariza* varieties).

Phenolic compounds are aromatic secondary metabolites ubiquitously spread through the plant kingdom comprising more than 8000 substances with highly diverse structures. Over the last few years, this family of compounds has caught a lot of attention due to the recognition of their antioxidant properties and their probable role in the prevention of several diseases [7,8,9,10], as well as their contribution to the organoleptic and functional properties in food products. Furthermore, the impact of these compounds in characterization, classification, and authentication studies cannot be underestimated. Polyphenolic distribution and content in natural food products seems to be related to food features such as the plant/fruit/seed variety, the geographical climate conditions of their production area, and the cultivation and manufacturing practices, between others. Consequently, phenolic profiling can be exploited as a source of analytical data to establish product classifications, as well as for the evaluation of food quality and the detection of adulterations [11,12].

Liquid chromatography (LC) in reversed-phase mode with UV detection or coupled to mass spectrometry (LC-MS) are among the most common techniques for the determination of polyphenols in a great variety of natural food products [13,14,15,16,17,18,19,20]. The use of polyphenolic profiles has also been described to achieve the characterization and classification of red paprika by means of chemometrics. For example, polyphenols and carbohydrates were employed as indicators of botanical and geographical origin of Serbian autochthonous clones of red spice paprika [16]. In relation with Spanish red paprika, a few works can be found dealing with the classification and authentication of this kind of samples. For example, geographical characterization of Spanish PDO paprika by multivariate analysis of multi-elemental content has been proposed [21]. Detection of adulterations in La Vera PDO paprika by free zone capillary electrophoresis (FZCE) [22,23] or by means of DNA typing methods [24] has also been described. However, to the best of our knowledge, phenolic profiling and HPLC-UV fingerprinting have not been yet described for that purpose.

In this work, a simple and feasible HPLC-UV method for the extraction of fingerprinting profiles based on phenolic compounds to achieve the classification of Spanish paprika samples was developed. For that purpose, a total of 17 phenolic compounds belonging to different families were selected and their separation by reversed-phase chromatography using a C_18_ column optimized. A simple and cheap sample extraction procedure based on sonication and centrifugation was employed. Extracting solution was selected according to the obtention of discriminant HPLC-UV fingerprints among the analyzed paprika samples. The hypotheses is that the data corresponding to the phenolic composition as well as the HPLC-UV chromatographic fingerprints can be considered as a source of potential descriptors to be exploited for the classification and characterization of Spanish red paprika samples by exploratory principal component analysis (PCA) and linear discriminant analysis (LDA) methods.

## 2. Materials and Methods

### 2.1. Chemicals and Standard Solutions

All reagents employed, unless otherwise stated, were of analytical grade. Standards of arbutine, resveratrol, ethyl gallate, syringaldehyde, polydatin, tyrosol, umbelliferon, 4-hydroxybenzoic acid, caffeic acid, chlorogenic acid, gallic acid, homogentisic acid, *p*-coumaric acid, vanillic acid, ferulic acid, syringic acid, and *trans*-cinnamic acid were obtained from Sigma-Aldrich (Steinheim, Germany). The structures and CAS numbers of all studied phenolic compounds are summarized in Table 1. Stock standard solutions of all phenolics (*ca.* 1000 mg/L) were prepared in methanol in amber glass vials. Intermediate working solutions were then prepared by appropriate dilution with Milli-Q water. All stock solutions were stored at 4 °C.

Methanol and acetonitrile (both UHPLC-gradient grade), absolute ethanol and acetone were purchased from Panreac (Barcelona, Spain). Water was purified using an Elix 3 coupled to a Milli-Q system (Millipore, Bedford, MA, USA) and filtered through a 0.22 µm nylon membrane integrated into the Milli-Q system.

### 2.2. Instrumentation

An Agilent 1200 Series HPLC instrument equipped with a G1311A quaternary pump, a G1322A degasser, a G1329A autosampler, and a G1314B ultraviolet-visible detector from Agilent Technologies (Palo Alto, CA, USA) was employed. Instrument was controlled with the Agilent ChemStation software. Chromatographic separation was performed in reversed-phase mode by using a Kinetex C_18_ (100 x 4.6 mm i.d., 2.6 µm particle size) column from Phenomenex (Torrance, CA, USA) at room temperature. Gradient elution employing 0.1% (*v*/*v*) formic acid aqueous solution (solvent A) and methanol (solvent B) as mobile phase components was applied following the next elution program: 0–2 min, isocratic step at 5% B; 2–4 min linear gradient from 5 to 25% B; 4–12 min, at 25% B; 12–14 min, from 25 to 45% B; 14–16 min, at 45% B; 16–18 min, from 45 to 95% B; 18–20 min, at 95% B; 20-21 min, back to initial conditions at 5% B; and from 21–30 min, at 5% B for column re-equilibration. The mobile phase flow rate was 1 mL/min and the injection volume was 20 µL. Direct UV-absorption detection at 280 nm was employed.

### 2.3. Samples and Sample Treatment

Two different scenarios were considered when purchasing the different paprika samples, viz. its region of production and its type. All paprika samples considered in this study were produced in Spain, either in the La Vera or Murcia regions, which are the two main producing regions in Spain. Furthermore, for each of the regions, the main types of paprika were considered; namely spicy and sweet, plus the bittersweet produced in La Vera. Overall, 96 Spanish paprika samples from different brands, purchased from different local shops and producers, were analyzed. Among them, 72 paprika samples were from La Vera PDO and belonging to three different varieties: sweet, bittersweet, and spicy, with 26, 23, and 23 samples, respectively, and 24 were from Murcia PDO and belonging to two varieties: sweet and spicy, with 12 samples each.

Sample treatment was performed as follows: 0.3 g of paprika sample was dispersed in 3 mL of a water:acetonitrile (20:80 *v*/*v*) solution in a 15 mL PTFE tube. Next, samples were stirred in a Vortex for 1 min (Stuart, Stone, United Kingdom) and sonicated for 15 min (2510 Branson ultrasonic bath, Hampton, NH, USA). Finally, samples were centrifuged for 30 min at 4500 rpm (Rotana 460 HR centrifuge, Hettich, Germany), and the supernatant extract filtered through 0.45 µm nylon filters (Whatman, Clifton, NJ, USA) and stored at –18 °C in 2 mL glass injection vials until analysis.

### 2.4. Data Analysis

Chemometric analysis of the chromatographic data was done in Matlab 7.1 (MathWorks, Natick, MA, USA) employing basic scripts written by the authors. Chromatograms were first baseline corrected and then compressed employing fast Fourier transform (FFT) to reduce its dimensionality while ensuring the preservation of the chromatographic profiles [25]. Calculated FFT coefficients were then analyzed by means of principal component analysis (PCA) and linear discriminant analysis (LDA) to assess the feasibility of the approach for the classification of paprika samples. The former allowed to reveal trends in the chromatographic responses, whereas the latter allowed to actually build a classification model. See further details in the Supplementary information file.

In this way, data matrices to be treated by PCA/LDA consisted on the whole HPLC-UV chromatographic fingerprint obtained at 280 nm.

## 3. Results and Discussion

### 3.1. HPLC-UV Method

The main objective of the present work was to develop a simple and non-expensive HPLC-UV method for the classification, characterization and authentication of Spanish paprika samples that can be feasible and easily accessible for any food control laboratory. To this aim, the first step was to achieve the separation of major phenolic compounds present in paprika, so that afterwards, when processing the whole chromatogram, differences in the chromatographic profiles (i.e., peaks and areas) of the different compounds are identified.

**Figure 1:**
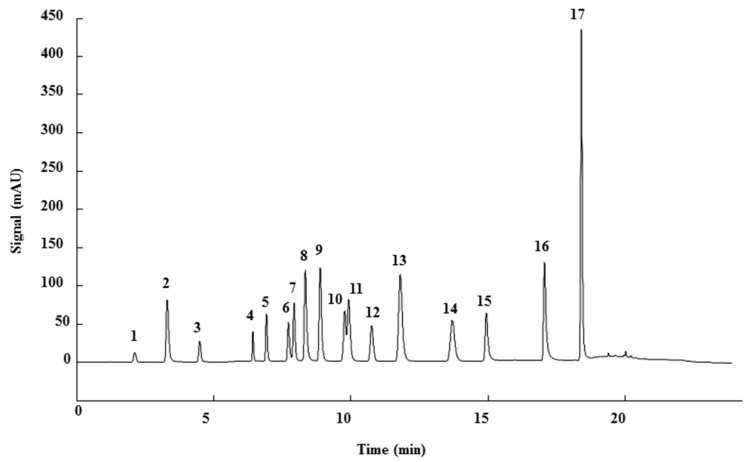
HPLC-UV chromatogram of a 15 mg/L standard solution of the 17 targeted phenolic compounds under optimal gradient elution conditions at 280 nm. Peak identification as in Table 1.

For that purpose, a total of 17 phenolic compounds belonging to different families (Table 1) were selected as target analytes, based on major phenolic compounds already identified in paprika, [16,26]. Their chromatographic separation was evaluated by reversed-phase chromatography using a porous-shell Kinetex C_18_ column. As it is well described in the literature, reversed-phase separation of phenolic compounds can be addressed with acidified aqueous solutions and methanol or acetonitrile as organic components in the mobile phase [20]. Thus, as a first approach, the separation of the 17 studied phenolic compounds was attempted employing 0.1% formic acid aqueous solution and methanol as mobile phase components, and using a universal gradient elution profile (from 5 to 95% methanol in 20 min, and then back to initial conditions for column re-equilibration). Under these conditions, peak signals for all the studied compounds were detected although full baseline separation for all of them was not achieved, and numerous partial co-elutions were observed. Hence, chromatographic separation was optimized by applying different gradient elution profiles combining methanol isocratic elution and linear gradient elution steps. Optimal gradient conditions (see Instrumentation section) were selected as a compromise between the separation of the targeted phenolic compounds and total analysis time. Figure 1 shows the HPLC-UV chromatogram obtained at 280 nm when a standard solution of targeted phenolic compounds (each at 15 mg/L) was analyzed under the optimized gradient program. As can be seen, the most critical phenolic pairs to be separated were chlorogenic acid/vanillic acid (peaks 6 and 7, respectively), and syringaldehyde/ethyl gallate (peaks 10 and 11, respectively). Achieving baseline separation of all compounds implied to considerably increase the total analysis time. For this reason, 20 min were considered as an acceptable limit to address the separation of the targeted phenolic compounds, taking also into consideration that the total analysis time will be higher as the column has to re-equilibrate at initial gradient conditions. Under these conditions, an acceptable separation for all compounds except for the pair syringaldehyde and ethyl gallate was obtained within 19 min, with a total chromatographic elution profile of 30 min.

Instrumental quality parameters of the proposed HPLC-UV method were calculated for the 17 phenolic compounds studied, and the figures of merit are summarized in Table 2. Instrumental limits of detection (ILODs), based on a signal-to-noise ratio of 3:1, were calculated using standard solutions prepared in Milli-Q water at low concentration levels, and values between 12.5 µg/L (vanillic acid) and 38.0 µg/L (arbutine) were obtained. Instrumental limits of quantitation (ILOQs), based on a signal-to-noise ratio of 10:1, in the range of 44.0 to 126.7 µg/L were achieved. In general, these values are slightly lower than the ones achieved with photo-diode array (PDA) detectors for these kind of compounds [27,28,29], which it is expected due to the characteristic improvement in sensitivity when employing monochromatic-UV detectors.

External calibration curves based on peak area within the concentration range from ILOQ to 50 mg/L were established for each phenolic compound, and very good linearities, with correlation coefficients (r^2^) values higher than 0.9996, were obtained. Sensitivity values, expressed as the slope of the calibration curves, between 6.29 (arbutine) and 138.66 (*trans*-cinnamic acid) were observed.

In order to evaluate the performance of the proposed HPLC-UV method both run-to-run (intra-day) and day-to-day (inter-day) precisions based on compound quantitation at several concentration levels (15, 2.5, 0.75, and 0.25 mg/L) were calculated and the results are also depicted in Table 2. With few variations depending on the concentration level evaluated, run-to-run precision values (calculated as percentage of relative standard deviation, RSD) were always lower than 0.7% (except for *trans*-cinnamic acid at 250 µg/L showing a RSD value of 2.7%). The dispersion increased for the day-to-day precision, as expected, with RSD values between 0.4 and 19.3%, and obtaining higher dispersion as the concentration level decreases.

Because of the lack of reference materials containing the 17 targeted phenolic compounds, intra-day (within the same day) method trueness was evaluated by comparing spiked phenolic compounds concentrations with the calculated ones using external calibration at the four concentration levels previously described, and the results expressed as the relative errors (%) are shown in Table 2. With some variations depending on the concentration level, the method trueness was, in general, very good with relative errors in the range from 0.04 to 8.9%, except for three compounds at the lowest concentration level (tyrosol, syringaldehyde, and *trans*-cinnamic acid) showing higher errors (13.3–29.2%).

The results obtained showed that the proposed HPLC-UV method is, in general, very satisfactory in terms of sensitivity, precision and trueness for the determination of phenolic compounds.

### 3.2. Sample Extraction Optimization

**Figure 2:**
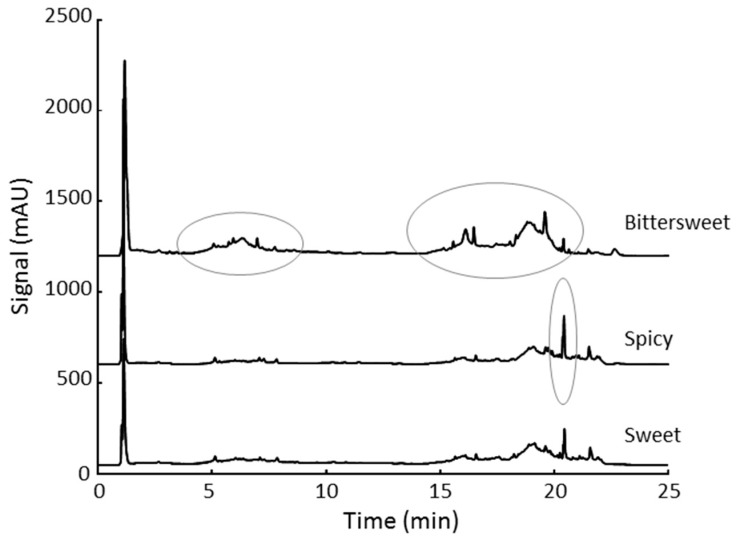
Representative chromatograms obtained for certain arbitrary paprika samples extracts.

The applicability of the proposed HPLC-UV method to obtain discriminant UV fingerprinting profiles for the classification and characterization of Spanish paprika samples was evaluated. For that purpose, a simple sample treatment procedure consisting in the extraction of 0.3 g of paprika sample with 3 mL of an extracting solvent by stirring, sonication and centrifugation was proposed. Several extraction solutions including 100% water, methanol, acetonitrile, ethanol and acetone, as well as the mixtures of water with the other organic solvents at 50:50, 20:80, and 80:20 *v*/*v* ratios, were employed. As a preliminary study, the proposed sample treatment using the different extracting solutions was applied to three paprika samples from La Vera PDO belonging to the same producer, but of different variety (sweet, bittersweet, and spicy). The obtained HPLC-UV chromatograms at 280 nm were compared in terms of signal abundance, signal profile, number of peaks detected, etc., in order to achieve discriminant fingerprints. The best results were obtained when the samples were extracted with a water:acetonitrile 20:80 *v*/*v* solution (see HPLC-UV fingerprints in Figure 2). As can be seen, noticeable differences in signal profiles and intensities were obtained in the time segments from 5 to 10 min and from 15 to 23 min, as well as a characteristic peak eluting close to 21 min whose intensity is clearly different depending on the paprika variety. A priori, we considered that these extracting conditions were appropriate to obtain sufficient discriminant HPLC-UV fingerprints for the characterization and classification of paprika samples. Hence, this extracting solution was selected, the proposed method applied to all the Spanish paprika samples, and the obtained HPLC-UV fingerprints subjected to chemometric analysis by PCA and LDA.

### 3.3. Qualitative Analysis

As already stated, our aim was to assess whether or not we can identify a pattern in the chromatographic profile that allows the classification of paprika samples based on its region and type. However, rather than trying to match each of the peaks observed on the chromatogram to a specific compound and analyze their areas, we aim to process the whole chromatogram (after its compression with FFT) as a unique profile and analyze it with the aid of pattern recognition methods such as PCA and LDA. Such a holistic approach based on the analysis of the patterns of several compounds has higher potential to address new challenges in food authentication than traditional quality control strategies focused on the analysis of a few compounds as there is not a unique marker that can be related to e.g., food origin.

Thus, the first step was the compression of the signals by means of FFT (see Supplementary information for further details). In this way, each chromatogram was reduced from 12,325 points down to 512 coefficients, without any loss of significant information (Figure S1). This was assessed by calculating both the correlation of determination (R^2^) and a comparison factor *fc* between the original and the reconstructed signal after doing the inverse of the FFT [25], with values higher than 0.99 for both of them.

**Figure 3:**
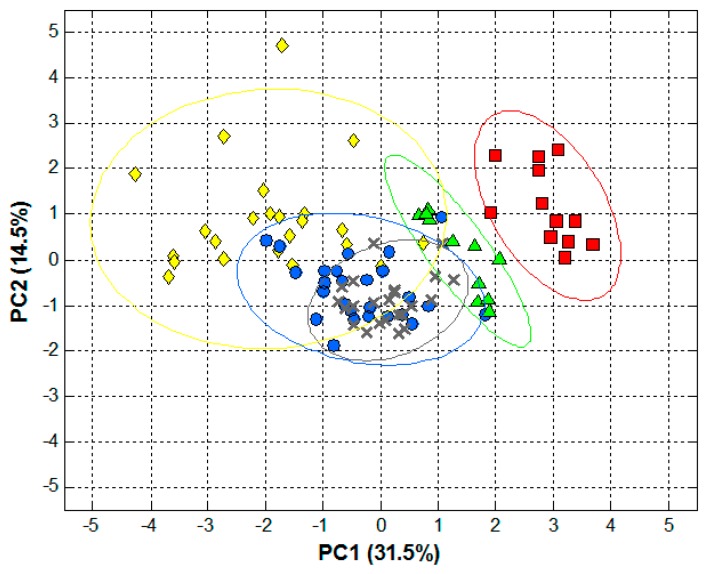
Score plot obtained after PCA analysis of the paprika samples chromatograms: (green **▲**) *Murcia* sweet, (red ■) *Murcia* spicy, (blue ●) *La Vera* sweet, (yellow **♦**) *La Vera* spicy, and (grey **x**) *La Vera* bittersweet. Ellipses plotted correspond to 95% confidence limits for each of the groups.

Next, PCA was used to assess initial patterns in the data as by plotting the PCs it provides a better representation of samples (dis)similarities, without taking into account prior expected similarities, but based only on their variance (Figure S2). With only the first two PCs, the cumulative variance was ca. 46.0%; meaning that almost half of the initial variance in the original data is now summarized on the plot shown in Figure 3.

Although not completely separated clusters were obtained for all the classes that we initially considered, we can still see some significant trends, e.g., the cluster of samples belonging to the Murcia spicy group. In order to further confirm these patterns, 95% confidence ellipses for each of the classes were also calculated and plotted [30], showing quite promising results taking into account that PCA is an unsupervised method that does not seek samples classification.

**Figure 4:**
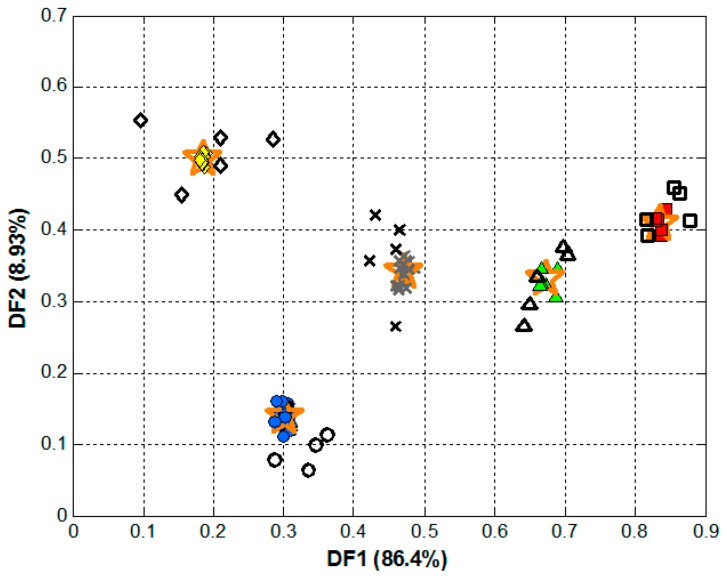
Score plot obtained after LDA analysis of the paprika samples chromatograms: (green **▲**) *Murcia* sweet, (red ■) *Murcia* spicy, (blue ●) *La Vera* sweet, (yellow **♦**) *La Vera* spicy, and (grey **x**) *La Vera* bittersweet. Additionally, the centroid for each of the classes is also plotted (★). Colored, filled symbols correspond to the samples of the training subset, black empty ones to the testing subset.

The next step was to build an actual model using a supervised method that allows the classification of samples into the different classes, to which aim LDA was the chosen tool (Figure S2). As before, chromatograms were baseline corrected and compressed with FFT, but in this case, calculated coefficients were then pruned employing a stepwise inclusion method in order to remove the less-significant coefficients [31]. The scores plot is shown in Figure 4, where clear clusters for each of the classes were obtained.

To numerically confirm the goodness of the classification model, the set of samples was randomly split into two subsets: training (71 samples) and testing (25 samples, 5 of each class). The former was used to build the classification model, providing the system with responses and target matrices to adjust the model parameters; whereas the latter was used to assess its predictive ability, providing the system only with responses matrix as input data to be interpolated in the built model and calculate the predicted targets matrix. This allows to obtain more unbiased metrics since the testing subset is not used at all during the modelling stage, and can therefore be considered as a blind set of samples, providing a measure of the actual performance of the classification model. Predicted and actual classes for each of the samples were compared, the confusion matrix was built (Table S1) and performance metrics were calculated, achieving 100% for classification rate, sensitivity and specificity [32]. Values that confirm the potential of HPLC-UV fingerprinting for the authentication of Spanish paprika samples, confirming the hypotheses established in the introduction section.

## 4. Conclusions

The combination of liquid chromatography with chemometric methods has been proven to be a suitable approach for the authentication of paprika samples, providing an analytical tool able to overcome the lack of knowledge of unique characteristic compounds that can be used to achieve such discrimination.

Extraction of phenolic compounds present in paprika was achieved by sonication/centrifugation, and its determination was carried out in reversed-phase chromatography using a C_18_ column. The developed method showed very good linearities, with slightly lower LOD and LOQ values than the ones reported with PDA detection. Run-to-run (intra-day) precisions, calculated as percentage of RSD were lower than 0.5% at 750 µg/L, whereas day-to-day precisions were around 2–10%. For the authentication of paprika samples, the registered chromatograms were compressed employing FFT and qualitative models built employing LDA, achieving the correct classification of all samples.

Overall, the main advantages of our approach are its simplicity, versatility, low-cost, and short-time analysis, all of them characteristics required to achieve the engagement of the stakeholders. Moreover, the approach developed herein can also be adapted to the discrimination of other solid food samples given the great relevance of phenolic compounds in plant products (e.g., fruits, vegetables, cereals, tea, or wine). In such cases, same extracting procedure and chromatographic conditions could be used as starting point given same target analytes would be considered. However, optimization of the chromatographic conditions, plus the analysis of a proper set of samples to build the chemometric model would still be required.

## Figures and Tables

**Table 1:** Chemical structures and classification of the studied phenolic compounds.

Peak	Phenolic compound	Family	Structure	CAS Number
1	Arbutine	Phenolic glucoside	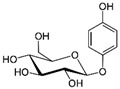	497-76-7
2	Gallic acid	Phenolic acid	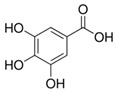	149-91-7
3	Homogentisic acid	Phenolic acid	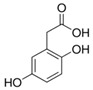	451-13-8
4	Tyrosol	Other phenolics	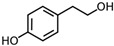	501-94-0
5	4-Hydroxybenzoic acid	Phenolic acid	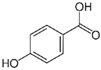	99-96-7
6	Chlorogenic acid	Phenolic acid	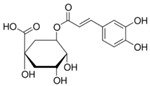	327-97-9
7	Vanillic acid	Phenolic acid	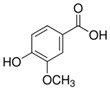	121-34-6
8	Caffeic acid	Phenolic acid	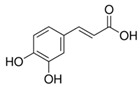	331-39-5
9	Syringic acid	Phenolic acid	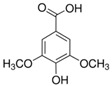	530-57-4
10	Syringaldehyde	Phenolic aldehyde	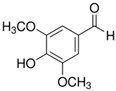	134-96-3
11	Ethyl gallate	Phenolic acid	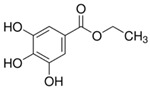	831-61-8
12	Umbelliferon	Coumarin	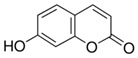	93-35-6
13	*p*-Coumaric acid	Phenolic acid	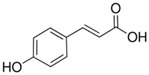	501-98-4
14	Ferulic acid	Phenolic acid	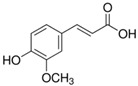	537-98-4
15	Polydatin	Stilben	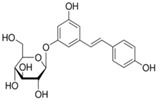	65914-17-2
16	Resveratrol	Stilben	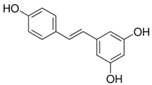	501-36-0
17	*trans*-Cinnamic acid	Cinnamic acid	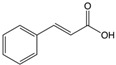	140-10-3

**Table 2:** Instrumental quality parameters of the proposed HPLC-UV method.

Peak	Polyphenol	ILOD (µg/L)	ILOQ (µg/L)	Linearity (r^2^)	Sensitivity		run-to-run precision (%RSD, n = 5)					day-to-day precision (%RSD, n = 3 × 5)					Trueness (% Error)			
							Level 1	Level 2	Level 3	Level 4		Level 1	Level 2	Level 3	Level 4		Level 1	Level 2	Level 3	Level 4
1	Arbutine	38.0	126.7	0.9996	6.29		0.1	0.1	0.1	0.4		0.4	3.9	1.4	19.3		0.5	0.7	3.1	7.9
2	Gallic acid	33.6	112.0	0.9997	36.70		0.1	0.05	0.4	0.3		0.4	2.7	2.1	10.7		1.0	0.7	1.6	2.5
3	Homogentisic acid	31.7	105.7	0.9996	11.50		0.1	0.2	0.4	0.4		1.8	4.5	9.1	17.2		1.0	0.3	0.4	5.1
4	Tyrosol	31.3	104.3	0.9996	9.07		0.1	0.1	0.1	0.7		2.1	3.3	4.7	9.1		1.0	0.2	5.3	13.3
5	4-Hydroxybenzoic acid	13.4	44.7	0.9996	18.05		0.04	0.1	0.1	0.2		1.8	3.3	3.7	10.0		1.0	1.3	4.7	4.3
6	Chlorogenic acid	33.3	111.0	0.9996	19.24		0.03	0.1	0.1	0.3		1.1	4.4	8.1	8.6		1.1	0.6	0.1	7.2
7	Vanillic acid	12.5	41.7	0.9999	26.39		0.1	0.1	0.2	0.3		0.8	3.2	3.3	13.5		0.04	1.0	4.6	1.0
8	Caffeic acid	13.2	44.0	0.9996	48.28		0.05	0.03	0.1	0.2		1.1	3.1	5.5	14.9		0.3	0.9	2.1	8.6
9	Syringic acid	12.8	42.7	0.9996	53.45		0.1	0.1	0.1	0.2		2.1	3.4	4.2	10.3		1.1	1.1	5.3	5.7
10	Syringaldehyde	31.0	103.3	0.9999	28.06		0.1	0.1	0.1	0.1		1.6	3.2	2.3	8.1		0.6	0.7	4.0	25.7
11	Ethyl gallate	13.0	43.3	0.9996	41.57		0.1	0.1	0.3	0.2		2.0	5.7	10.8	14.3		1.0	3.4	0.5	1.3
12	Umbelliferon	31.2	104.0	0.9996	25.50		0.1	0.03	0.1	0.5		1.1	3.8	5.9	17.7		0.3	0.2	2.4	8.9
13	*p*-Coumaric acid	14.3	47.7	0.9999	75.10		0.1	0.2	0.1	0.1		1.2	4.2	6.0	13.6		0.8	1.1	0.1	4.4
14	Ferulic acid	31.1	103.7	0.9999	46.79		0.1	0.04	0.1	0.2		1.4	3.9	6.3	14.9		0.9	0.9	1.6	5.2
15	Polydatin	33.0	110.7	0.9999	37.26		0.1	0.2	0.5	0.4		1.4	2.5	7.9	13.7		0.5	2.0	0.6	8.7
16	Resveratrol	31.1	103.7	0.9999	63.62		0.04	0.2	0.3	0.4		1.4	4.5	8.2	18.3		1.2	0.6	4.6	3.7
17	*trans*-Cinnamic acid	12.6	42.0	0.9999	138.66		0.1	0.1	0.4	2.7		3.3	2.7	5.6	8.1		1.4	0.5	8.3	29.2

Level 1 = 15 mg/L; Level 2 = 2.5 mg/L; Level 3 = 750 µg/L; Level 4 = 250 µg/L.

## Supplementary Materials

This supplementary provides further description of the fingerprinting approach, namely about the signal compression method used (fast Fourier transform, FFT) and the chemometric analysis (both principal component analysis (PCA) and linear discriminant analysis (LDA)). The following are available online at http://www.mdpi.com/1424-8220/18/12/4479/s1, Figure S1: Selection of the optimal number of Fourier coefficients for signal compression. Changes in (A) chromatogram reconstruction and (B) R^2^ and *fc* coefficients depending on the number of coefficients considered; Figure S2. Schematic comparison between PCA and LDA. Briefly, PCA seeks for the direction of maximum variance between the variables, while LDA seeks for the direction that provides maximum separation between the classes. Note also that in PCA as many new PCs as variables in the original data are obtained, whereas in LDA only as many #groups-1 DFs are obtained. Adapted from Cetó, X., et al. Electroanalysis, 25 (2013), 1635; Table S1. Confusion matrix built according to the classes assigned by the LDA model to the samples of the testing subset.
