# Supplementary material for: Determination of HPLC-UV Fingerprints of Spanish Paprika (Capsicum annuum L.) for Its Classification by Linear Discriminant Analysis
Source: Sensors (Basel). 2018 Dec 18;18(12):4479. doi: 10.3390/s18124479 (PMC6308838; doi:10.3390/s18124479)
Supplement: Supplementary file 1 [file sensors-18-04479-s001.pdf]

Determination of HPLC-UV Fingerprints of Spanish Paprika (*Capsicum annuum* L.) for its Classification by Linear Discriminant Analysis

Xavier Cetó [1], Núria Serrano [1,*], Miriam Aragó [1], Alejandro Gámez [1], Miquel Esteban [1], José Manuel Díaz-Cruz [1] and Oscar Núñez [1,2,3]

[1] Department of Chemical Engineering and Analytical Chemistry, University of Barcelona. Martí i Franquès 1-11, E08028 Barcelona, Spain.
[2] Research Institute in Food Nutrition and Food Safety, University of Barcelona. Av. Prat de la Riba 171, Edifici Recerca (Gaudí), E-08901 Santa Coloma de Gramanet, Barcelona, Spain.
[3] Serra Hunter Fellow. Generalitat de Catalunya, Spain.
* Correspondence: nuria.serrano@ub.edu; Tel.: 34-93-403-3706

Supporting Information

This supplementary provides further description of the fingerprinting approach, namely about the signal compression method used (fast Fourier transform, FFT) and the chemometric analysis (both principal component analysis (PCA) and linear discriminant analysis (LDA)).

Fast Fourier transform

In order to reduce the large dimensionality of the chromatograms, FFT algorithm was used. Fourier transform could be considered as the decomposition of the signal using a sine/cosine function pair at different frequencies, calculating a coefficient for each one taking into account its contribution to the original signal. In this way, the chromatogram was decomposed into components of different frequencies. Next, higher frequency components can be discarded as those are mainly related to noise, whereas most relevant information is kept in the few first coefficients.

Selection of the number of coefficients can be carried by applying the inverse Fourier transform and evaluating signal reconstruction. For this purpose, two different metrics were considered: correlation of determination (R^2) and the comparison factor *fc*. The former is calculated from the linear regression point-to-point between the original and reconstructed signals, whereas the latter is defined as the ratio of the area under the intersection of both signals to the total area under both curves: $fc = (A \cap B) / (A \cup B)$. Both factors range from 0 (complete lack of similarity between signals) to 1 (signals are identical), and increase

exponentially as similarity does. However, the usage of *fc* is recommended as it is more sensitive to small differences (Fig. S1).

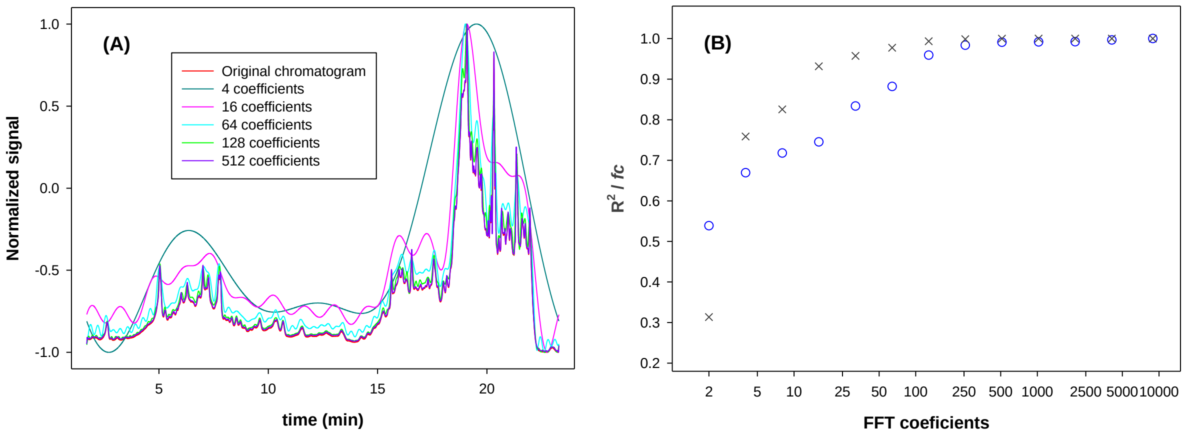

Fig. S1. Selection of the optimal number of Fourier coefficients for signal compression. Changes in (A) chromatogram reconstruction and (B) R^2 and *fc* coefficients depending on the number of coefficients considered.

Principal component analysis and Linear discriminant analysis

PCA is a mathematical procedure that allows the projection of the information carried by the original variables onto a smaller number of underlying variables called principal components (PCs) through an orthogonal transformation. These PCs are calculated on the basis of the maximum explained variance. Then, by plotting the PCs, it is possible to obtain information about the interrelationships between different samples and variables and detect and interpret sample patterns, groupings or (dis)similarities.

LDA is a supervised classification method based on the Bayes formula that constructs a predictive model to evaluate group membership of a sample. In this case, these new variables are called discriminants functions (DF's) and based on linear combinations of the predictive variables that provide the best discrimination between the groups.

Although both are quite similar in the sense that they do look for linear combinations of variables which best explain the data, there is a significant difference between PCA and LDA (Fig. S2). While PCA is an unsupervised method that does not take into account any difference between classes, LDA is a supervised method that explicitly attempts to model the difference between the classes of data. Therefore, PCA provides just a visualization of samples (dis)similarities, but not implying any clustering, whereas LDA actually builds a classification model that allow its classification.

Additionally, to further improve the performance of the LDA model, a stepwise inclusion method was used to further remove the variables (FFT coefficients in this case) that do not contribute to the classification task. Inclusion criteria was based on the maximization of Mahalanobis distance between groups, until a maximum was reached. In this way, less significant variables were not further considered during the modelling, so as to have a simpler model, which in turn improves its generalization ability.

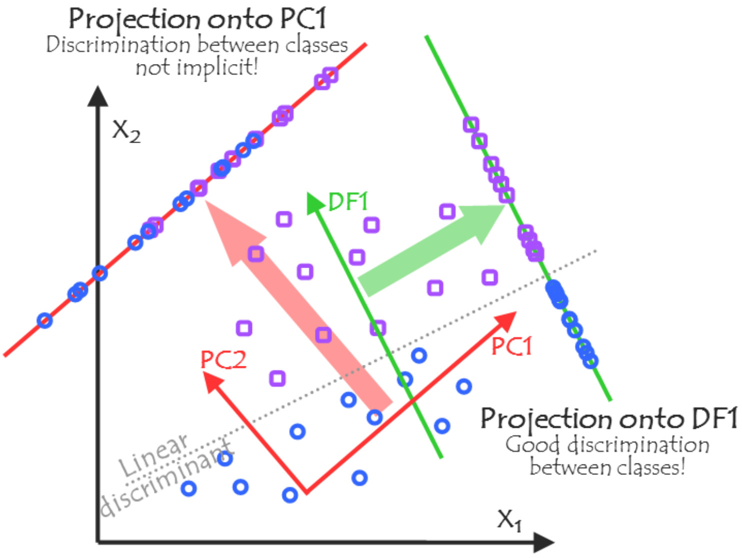

Fig. S2. Schematic comparison between PCA and LDA. Briefly, PCA seeks for the direction of maximum variance between the variables, while LDA seeks for the direction that provides maximum separation between the classes. Note also that in PCA as many new PCs as variables in the original data are obtained, whereas in LDA only as many #groups-1 DFs are obtained. Adapted from Cetó, X., *et al.* Electroanalysis, 25 (2013), 1635.

Table S1. Confusion matrix built according to the classes assigned by the LDA model to the samples of the testing subset.

	V Sp [b]	*V Sw* [b]	*V Bs* [b]	*M Sp* [b]	*M Sw* [b]
V Sp [a]	5	0	0	0	0
V Sw [a]	0	5	0	0	0
V Bs [a]	0	0	5	0	0
M Sp [a]	0	0	0	5	0
M Sw [a]	0	0	0	0	5

[a] Expected; [b] Found. M: *Murcia*; V: *La Vera*; Sw: *sweet*; Bs: *bittersweet*; Sp: *spicy*.
